# The Prevalence and Nature of Eating and Swallowing Problems in Adults with Fibromyalgia: A Systematic Review

**DOI:** 10.1007/s00455-023-10597-8

**Published:** 2023-06-22

**Authors:** Órla Gilheaney, Andrea Chadwick

**Affiliations:** 1 Department of Clinical Speech & Language Studies, School of Linguistic, Speech & Communication Sciences, Trinity College Dublin, Room 102, 7-9 South Leinster Street, Dublin, Ireland; 2 University of Kansas Medical Center, 10710 Nall Ave, Overland Park, KS 66211 USA

**Keywords:** Dysphagia, Fibromyalgia, Pain, Swallowing, Prevalence

## Abstract

Fibromyalgia is a complex chronic pain condition characterized by widespread pain, fatigue, cognitive dysfunction, and sleep disturbances. People with fibromyalgia can experience both autonomic and somatic disturbances, cognitive and mental health symptoms, and hypersensitivity to external stimuli. Fibromyalgia often co-occurs with a range of well-researched comorbidities (e.g., temporomandibular disorders, migraine, and irritable bowel syndrome). However, emerging research suggests that individuals with fibromyalgia also often experience eating, drinking, and swallowing problems (e.g., odynophagia, glossodynia, etc.). However, there is very little known about these issues, their psychosocial impact, or the best means of managing them clinically. As such, the aim of this research was to examine the epidemiology, prevalence and nature of eating and swallowing problems in adults with fibromyalgia as reported within previous research. A systematic search of electronic databases, selected conference proceedings, and reference lists was completed in March 2021, with no date or language restrictions. Studies reporting the presence and nature of eating and drinking problems in this cohort were included. Eligibility was assessed by two independent reviewers who also critically appraised the included studies using the Joanna Briggs Tool. This literature search yielded a total of 38 potentially eligible studies, with 6 studies included in analysis. Studies were highly heterogeneous in methodology and design, with meta-analysis showing that dysphagia and GERD are prevalent in fibromyalgia patients (51.9% and 25.9%, respectively), among other issues. From review of existing literature, eating and swallowing problems appear to be common among adults with fibromyalgia, with potential additional repercussions for activity, participation, and quality of life. Further research is required to prospectively investigate these issues, with patient and public involvement necessary to guide impactful research planning.

## Introduction

Fibromyalgia (FM) is a chronic pain disorder where the hallmark symptom is widespread and diffuse pain, with common associated symptoms including sleep disturbances, cognitive dysfunction, fatigue, headaches, depression and anxiety, and other co-occurring pain syndromes [1]. The global prevalence of fibromyalgia ranges from 2 to 8% within the general population [1–5]. There are a range of diagnostic criteria used globally, with subsequent varied nomenclature, essential criteria for diagnosis, and means of assessment. This has led to difficulties in standardized diagnosis and treatment which can complicate the provision of effective management. Following newer diagnostic criteria that was released in 2017, the disease has a female:male ratio of 2:1, similar to other pain conditions [6]. The prevalence is similar in different countries, cultures, and ethnic groups and there is no evidence that FM has a higher prevalence in industrialized countries and cultures [1, 5].

The current understanding of the pathophysiology of FM is that it is thought of as a “centralized pain” state, where amplification of pain perception is largely driven by changes within the central nervous system (central sensitization) [7, 8]. Central sensitization is defined as “an amplification of neural signaling within the central nervous system that elicits pain hypersensitivity” [8]. It is hallmarked by the presence of allodynia, a painful response to a stimulus that is usually considered “non-noxious”, and/or hyperalgesia, which is an increased pain response to a noxious stimulus. The mechanisms underlying central sensitization are not fully established, but it is likely that there are both peripheral and central components that play a role in this phenomenon that is seen in centralized pain disorders. Central sensitization could potentially originate in the periphery and through a variety of mechanisms that could eventually lead to long-term potentiation in the spinal cord as well as structural changes in the brain. These central nervous system changes are then the key to the maintenance of increased pain perception, with structural and functional MRI brain scans of patients with fibromyalgia demonstrating associated changes in the volume of gray matter, reduced connectivity in descending pain-modulating pathways, and increased activity and connectivity within “pro-pain” regions of the brain [9, 10]. Beyond pain, co-existing somatic symptoms including memory difficulties, fatigue, and sleep disturbances as well as cognitive/affective symptoms (e.g., catastrophizing, anxiety, depression) are frequently observed [11, 12].Given this broad range of difficulties within both the CNS and its peripheral components, authors here hypothesize that it is possible that the complex neurological control of the swallow mechanism may also potentially be disrupted, leading to overall eating and drinking problems, including motility difficulties, pain on mastication or swallowing, or other sensorimotor abnormalities.

The prognosis for recovery in FM using traditional medicine is generally poor [13]. Traditional therapies include pharmacologic interventions including anti-depressants, non-steroidal anti-inflammatory drugs, muscle relaxants, and anti-epileptics [14]. The results of these interventions are only modestly effective as pharmacologic interventions have been reported to achieve symptom relief in approximately only a third of patients in the FM population [15, 16].

Patients with FM commonly have lifelong histories of chronic pain throughout their body and many chronic pain syndromes tend to co-occur with FM including migraine, temporomandibular joint disorder (TMD), irritable bowel syndrome, endometriosis, and urologic chronic pelvic pain syndrome (UCPPS) [17]. The pain associated with FM may lead to decreased physical strength and functioning, job loss, and in some, difficulties in meeting basic routine daily needs [18], such as the need to hydrate and nourish oneself adequately. Abnormalities with swallowing, known in medical terminology as dysphagia, are not officially classified as a feature of FM nor is it part of the current diagnostic criteria for FM, however, has been reported as a common patient experience within some recent studies [19–24]. In addition, there are significant amounts of anecdotal patient reports available in online forums which reference a myriad of eating and drinking difficulties (e.g., pain on swallowing, the sensation of a bolus sticking in the throat, impaired mastication; altered taste, intra-oral burning, or dry mouth). These anecdotal accounts tentatively suggest wider repercussions beyond the sensorimotor process of swallowing, such as unintentional weight changes and alterations in oral intake and dietary habits, in line with reports from other clinical cohorts. However, despite these patient reports, there is very little known academically to fully describe and quantify these eating and drinking issues, their potential repercussions, their psychosocial impact, or the best means of managing them clinically.

This lack of research information results in working clinicians being unsure of the existence or significance of dysphagia among adults with fibromyalgia, which can lead to a lack of validation of the patients’ experience or priorities, inappropriate management of such issues, and poor patient outcomes and experience of care. As such, the following primary research question guided the conduct of this study: “what is the epidemiology of eating and swallowing problems in adult patients presenting with fibromyalgia?”. From this main question, 2 sub-questions emerged: “What is the prevalence of eating and swallowing problems in adult patients presenting with Fibromyalgia?” and “what is the nature of eating and swallowing problems in adult patients presenting with Fibromyalgia?”.

The aim of this research was therefore to examine the epidemiology, prevalence and nature of eating and swallowing problems in adults presenting with fibromyalgia as reported within previous research.

Studies were included if they provided data on primary or secondary outcomes. It was intended that this study would act as the first step in understanding these issues, paving the way for future prospective, patient-led research on the topic, with the ultimate goal of improved patient care delivery and lived experience.

## Methods

The Preferred Reporting Items for Systematic Reviews and Meta-Analyses statement (PRISMA) [25] informed the conduct of this research. The protocol for this review was prospectively published on the Prospero database [CRD42021240139] [26].

### Outcomes of Interest

The outcomes of interest investigated here were based on both specific signs of eating and swallowing difficulties commonly discussed within the general dysphagia literature and also issues which are frequently reported within patient accounts: Primary outcomes of interest included those discussed as most common and most impactful on overall health and well-being, such as:

- Dysphagia or impaired swallowing as reported subjectively and/or investigated using clinical examination, investigator-reported outcome measures, patient interviews or questionnaires, and/or standardized imaging techniques;
- Impaired mastication as reported subjectively and/or investigated using clinical examination, investigator-reported outcome measures, patient interviews or questionnaires, and/or standardized imaging techniques;
- Pain on chewing as reported via patient interviews or questionnaires, or subjective scales;
- Fatigue on chewing as reported via patient interviews or questionnaires, or investigated using clinical examination, or EMG assessment; and
- Aspiration/penetration on food/liquids/secretions as investigated using clinical examination, investigator-reported outcome measures, and/or standardized imaging techniques.

Secondary outcomes included secondary or additional issues, such as:

- Odynophagia as reported subjectively and/or investigated using clinical examination, investigator-reported outcome measures, patient interviews or questionnaires;
- Xerostomia as reported subjectively and/or investigated using clinical examination, investigator-reported outcome measures, patient interviews or questionnaires;
- Glossodynia as reported subjectively and/or investigated using clinical examination, investigator-reported outcome measures, patient interviews or questionnaires;
- Dysgeusia as reported subjectively and/or investigated using patient interviews or questionnaires;
- Sensation of choking/bolus sticking in pharyngeal region as reported subjectively and/or investigated using patient interviews or questionnaires;
- Unintentional weight loss as reported by patients and/or investigated via clinical examination; and
- Gastroesophageal Reflux Disease (GERD) as reported subjectively and/or investigated using clinical examination, investigator-reported outcome measures, patient interviews or questionnaires, and/or standardized imaging techniques.

### Eligbility Criteria

#### Studies

Eligible studies included:

- Published and unpublished research in using a randomized and non-randomized, retrospective, cross-sectional, or observational design investigating the prevalence and nature of eating and drinking problems in adults diagnosed with fibromyalgia (as diagnosed clinically or reported by patient themselves) were eligible for inclusion.
- There were no language, geographic, study design, or date limitations applied.

#### Participants

Eligible participants included:

- Adults diagnosed with FM (as diagnosed clinically or reported by patient themselves) attending any healthcare setting

There were no other sex/severity/illness duration limitations imposed. Studies were excluded if participants were not adults diagnosed with FM or if they had a history of head and neck cancer, head and neck surgery, and/or neurological conditions affecting this area.

### Information Sources

A systematic search which encompassed search filters, medical subject headings, and key-text terms was developed for use here (see Supplemental Materials for full replicable search string). The databases which were independently searched by both authors from inception to March 2021 were: PubMed, CINAHL, Web of Science, and The Cochrane Database of Systematic Reviews. No search limits regarding publication date, language, or location of publication were applied here.

Independent hand-searches of the proceedings of the annual scientific meetings of the European Society for Swallowing Disorders (2011–2021) and the Dysphagia Research Society (both published in *Dysphagia*) (1992–2021) were conducted. Hand-searches of the bibliographies of studies which were ultimately included were also conducted to identify records not indexed in the directories initially searched or not identified by the initial search.

Authors of primary studies were contacted if researchers were unable to access potentially eligible articles or if there was missing data within articles which were published within the last 10 years. Initial contact was conducted via email using a standardized email template, with a reminder email after 2 weeks if there was no response, and subsequent exclusion of the article if no response was received to 2 emails.

### Study Selection

Records identified through the systematic search were initially exported to Zotero [27], with subsequent transference to Covidence [28] for automatic removal of duplicates. Authors then independently screened all remaining records at title/abstract level, with progression of appropriate records to the full-text screening stage if they met eligibility criteria. Any inconsistencies between authors regarding eligibility were initially flagged by Covidence and discussed, with any ongoing disputes mediated by a third independent reviewer. Inclusion/exclusion of records was tracked by automatic generation of a PRISMA flow diagram by the Covidence platform.

### Data Collection Process and Data Items

Data was independently extracted by both authors using the Joanna Briggs Institute (JBI) prevalence data extraction form [29] on topics such as: methodological details, recruitment, sampling, demographics, outcome measurement, and prevalence data. Data were then cross-checked for concordance, with another independent reviewer available to mediate disputes, if required, however, as all data extraction was consistent, this option was not required. Data were extracted on the primary and secondary outcomes of interest listed above.

### Critical Appraisal of Included Studies

To critically appraise individual studies, the first author independently utilized the JBI Checklist for Prevalence Studies [30]. This tool is readily accessible online and facilitates the critical appraisal of the methodological quality of a study to inform the interpretation of individual study results while also providing information to guide future syntheses [30]. The inclusion/exclusion of a range of methodological factors are scored (e.g., sampling frame, study size, measurement of the condition in a standard and reliable way) as per “yes” (2 points)/”no” (0 points)/”unclear” (1 point)/”not applicable”. In this study, quality bands were established as follows: poor quality = 0–6 points; moderate quality = 7–12 points; strong quality = 13–19 points. Risk of bias across studies was mitigated for as follows: (1) publication bias was addressed by designing a systematic search strategy which accounted for electronic databases, grey literature, and results not indexed in traditional methods; (2) selective reporting within studies was addressed by contacting authors were data was inconsistent/missing and seeking further information.

### Summary Measures and Synthesis of Results

Narrative description of included studies was conducted, with descriptive statistics used to initially explore data. Meta-analysis was conducted using the CMA Software programme [31]. Prevalence figures were presented using 95% CIs, with forest plots constructed for all estimates. Data were pictographically displayed using accessible graphs and charts to visually synthesize findings.

## Results

**Fig. 1:**
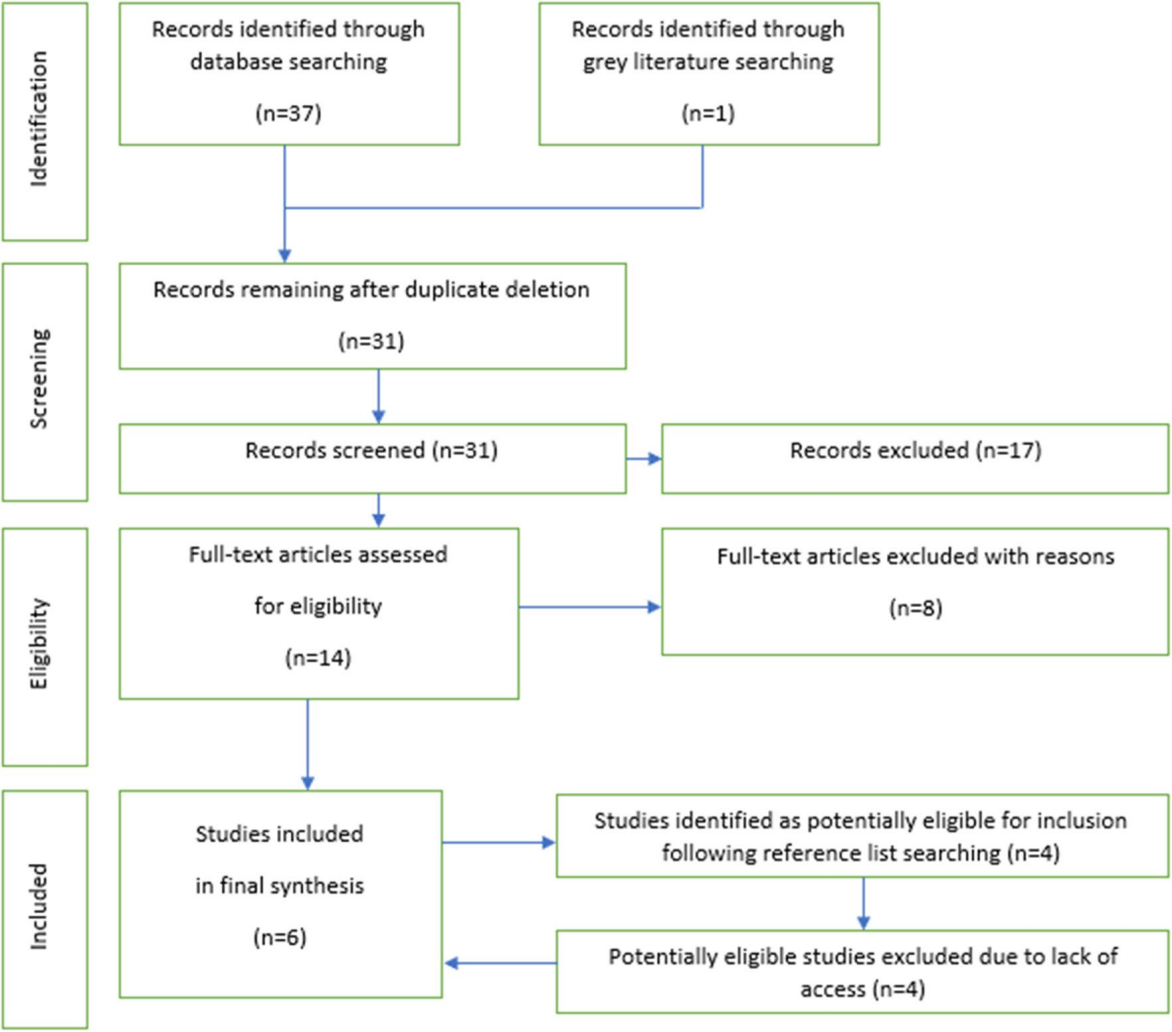
PRISMA Diagram

A total of 37 results were retrieved during the initial database search (see Fig. 1 and Table 1), with 1 subsequent record identified as potentially eligible for inclusion from the Dysphagia Research Society conference proceedings in 2020. As such, 38 potentially eligible records were initially identified. Following screening, 6 studies were ultimately included (Fig. 1).

**Table 1:** Results from database searches

Database searched	Potentially eligible studies identified
PubMed	13
Web of science	16
Cochrane database of systematic reviews	5
CINAHL	3

### Study Characteristics

#### Participant Demographics

Data regarding 2927 individuals with fibromyalgia were extracted. As outlined in Table 2, the pooled age range and gender ratio were unspecified in the majority of studies.

**Table 2:** Participant demographics

Citation	No. of patients with Fibromyalgia studied	Gender details	Mean age (SD) of patients (years)	Mean age of onset (years)	Mean disease duration (SD) (months)
Jalilvand et al. [20]	27	Gender unspecified for fibromyalgia group specifically	Unspecified	Unspecified	Unspecified
Colón León & Centeno Vázquez, [24]	131	Females = 124; Males = 7	Unspecified	Unspecified	Unspecified
Seccia et al. [23]	1	Females = 1; Males = 0	80	80	Unclear
Rhodus et al. [21]	67	Females = 67; Males = 0	Unspecified	Unspecified	Unspecified
Bhadra & Petersel [19]	2624	Unspecified	Placebo group (*n* = 689) = 48.9 (11.2) Pregabalin 300 mg/day group (*n* = 686) = 48.9 (10.7) Pregabalin 450 mg/day group (*n* = 686) = 48.9 (11.3) Pregabalin 600 mg/day group (*n* = 563) = 49.7 (11.2)	Unspecified	Placebo group (*n* = 689) = 110.7 (95.5) Pregabalin 300 mg/day group (*n* = 686) = 105.7 (92.6) Pregabalin 450 mg/day group (*n* = 686) = 110.2 (97.5) Pregabalin 600 mg/day group (*n* = 563) = 115.3 (99.4)
Piersiala et al. [22]	77	Gender unspecified for fibromyalgia group specifically	Unspecified	Unspecified	Unspecified

#### Characteristics of Included Studies

All included studies were published in English. Four of the six studies intentionally investigated dysphagia in those with fibromyalgia as a primary aim [21–24]. Study designs varied, including retrospective cohort studies (*n* = 2) [20, 22] one cross-sectional study [24] one post hoc analysis (*n* = 1) [19], one case–control study [21], and one case study [23]. Studies were primarily conducted in the USA (*n* = 3; 50%) [19, 21, 22], with 2 studies providing unclear details regarding specific location (33.3%) [20, 24], and 1 study published in Italy (16.6%) [23]. Study settings were as follows: medical or surgical hospital clinics (*n* = 2) [21, 22], emergency departments (*n* = 2) [23, 24], support groups and social networking sites [24]. One study recruited from phase III pharmacological trials [19], while one further study [20] was unclear regarding the specific setting. Data collection tools were variable (see Table 3).

**Table 3:** Characteristics of included studies

Citation	Year of publication	Study aims	Study design	Region from which participants were recruited	Setting from which participants were recruited	Ethical approval granted	Sampling procedure	Recruitment procedure
Piersiala et al. [22]	2020	To investigate whether the clinical presentation of fibromyalgia, irritable bowel syndrome, and chronic fatigue syndrome is similar in a population presenting with voice and laryngeal disorders	Retrospective cohort study	USA	Retrospective review of notes in a hospital facility	Waived informed consent due to retrospective nature	Retrospective sampling of notes during specific time frame	Retrospective
Bhadra and Petersel, [19]	2010	To analyze the prevalence of comorbid conditions and relationship to pregabalin efficacy in patients with fibromyalgia pooled from 4 Phase III clinical trials	Post hoc analysis of multiple pooled RCT	USA	Multiple settings: post hoc analysis of previously collected RCT data	Yes, for studies included in the post hoc analysis	Purposive sampling from four previously conducted double-blind, randomized, placebo controlled studies	Retrospective recruitment from studies previously conducted
Jalilvand et al. [20]	2019	To compare preoperative symptomology and objective work-up and patient reported outcomes of GERD symptom control and disease-specific quality of life in patients with and without baseline irritable bowel syndrome/fibromyalgia diagnoses following laparoscopic Nissen fundoplication	Retrospective cohort study	Not stated	Not specified—all patients recruited from "a single setting"	Not stated	Unclear	Unclear
Colón León and Centeno Vázquez [24]	2021	To prove if Hispanic patients diagnosed with fibromyalgia could be presenting concomitant symptoms of dysphagia and to describe a possible relationship between both conditions and its symptoms	Cross-sectional survey	Hispanic countries (unclear)	Multiple settings: medical offices, fibromyalgia support groups and via social networks in Hispanics countries	Not stated	Not stated	Not stated
Seccia et al. [23]	2015	To report on the case of a patient with fibromyalgia, who presented with dysphagia, odynophagia, and glossodynia	Case study	Italy	Emergency department	Not stated, states informed consent obtained	Convenience sampling	The patient was not explicitly asked for the consent for inclusion in this study as the authors state that she cannot be identified from the report
Rhodus et al. [21]	2003	To evaluate the prevalence and profile of various oral symptoms in a population of patients diagnosed with fibromyalgia	Case–control study	USA	University rheumatology clinic	Yes	Convenience sampling	Not stated

### Results of Individual Studies

#### Synthesis of Results

##### Primary Outcomes

**Fig. 2:**
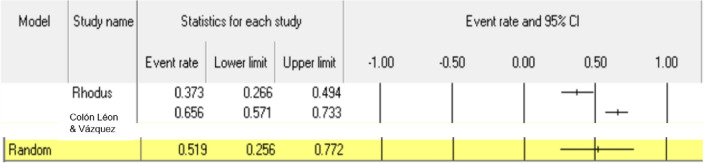
Forest plot regarding prevalence of dysphagia

Primary outcomes were under-reported in included studies, rendering it difficult to sufficiently answer research questions. Dysphagia was reported in 3 studies [21, 23, 24] Only two studies [21, 24] could be included in meta-analysis as Seccia only recruited 1 participant [23]. This prevalence was estimated on random-effects meta-analysis to be 51.9% (95% CI: 25.6–77.2%) (see Fig. 2).

Aspiration/penetration on food/liquids/secretions or impaired mastication or pain or fatigue on chewing were not reported in any studies Table 4.

**Table 4:** Results of individual studies

Citation	Data sources	Prevalence of dysphagia	Prevalence of fatigue on chewing	Prevalence of impaired mastication	Prevalence of pain on chewing	Prevalence of aspiration/penetration on food/liquids/secretions	Prevalence of odynophagia	Prevalence of xerostomia	Prevalence of glossodynia	Prevalence of dysguesia	Prevalence of sensation of choking/bolus sticking in pharyngeal region	Prevalence of unintentional weight loss	Prevalence of GERD
Piersiala et al. [22]	Collection of demographic and medical data from medical charts during the study period	No data available	NM	NM	NM	NM	NM	NM	NM	NM	NM	NM	19.5% (*n* = 15)
Bhadra and Petersel [19]	Subjective assessment/ patient report questionnaires: daily pain diaries; self-reported presence of gastrointestinal symptoms; unspecified questionnaires regarding medical histories; The Short-Form McGill Pain Questionnaire[32]; a patient global impression of change assessment Clinical examination: The American College of Rheumatology criteria for diagnosing fibromyalgia [33]	NM	NM	NM	NM	NM	NM	NM	NM	NM	NM	NM	26.02% (*n* = 683)
Jalilvand et al. [20]	Objective testing: unspecified forms used to collect data from database regarding objective testing (eg: endoscopic dilation post-operatively) Subjective assessment/ patient report questionnaires: unspecified forms used to collect data from database regarding demographics, medication use, and symptoms; GERD-HRQL [34]	NM	NM	NM	NM	NM	NM	NM	NM	NM	48% (*n* = 13)	NM	NM
Colón León & Centeno Vázquez [24]	Subjective assessment/ patient report questionnaires: EAT-10 scale [35]; unspecified demographic questionnaire	66% (*n* = 86)	NM	NM	NM	NM	NM	NM	NM	NM	NM	NM	NM
Seccia et al. [23]	Objective testing: blood pressure and heart rate monitoring; blood testing; EKG; X-Ray; transthoracic echocardiogram; esophagogastroduodenoscopy Subjective assessment/ patient report questionnaires: case history; Widespread pain index and symptom severity scale [33] Clinical examination: The American College of Rheumatology criteria for diagnosing fibromyalgia [33]	100% (*n* = 1)	NM	NM	NM	NM	100% (*n* = 1)	NM	100% (*n* = 1)	100% (*n* = 1)	NM	NM	NM
Rhodus et al. [21]	Subjective assessment/ patient report questionnaires: Xerostomia questionnaire [36, 37] Melzack-McGill Pain Scale [38]; unspecified taste and swallowing questionnaire; unspecified oral symptoms questionnaire; Rheumatic Problems Questionnaire (of the University of Minnesota TMJ Clinic) Clinical examination: The American College of Rheumatology criteria for diagnosing fibromyalgia [33]; unspecified oral examination	37.3% (*n* = 25)	NM	NM	NM	NM	NM	70.9% (*n* = 48)	32.8% (*n* = 22)	34.2% (*n* = 23)	NM	NM	NM

*NM*  not measured

##### Secondary Outcomes

**Fig. 3:**
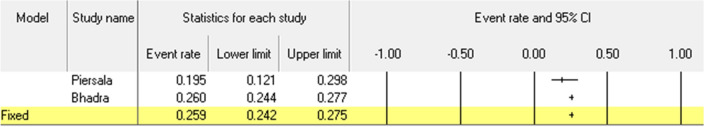
Forest plot regarding prevalence of GERD

GERD was reported in 2 studies (*n* = 760) [19, 21], with this prevalence estimated to be 25.9% (95% CI: 24.2–27.5%) (see Fig. 3).

As above, minimal reporting of the secondary outcomes was found within included studies, hindering authors in sufficiently answering research questions. Meta-analysis could not be completed for the outcomes of glossodynia or dysgeusia due to discrepancies in sample size and study design. Narratively, glossodynia was reported in 2 studies [21, 23], however. Rhodus [21] reported glossodynia in 32.83% of participants, while Seccia’s [23] one participant experienced this issue. Dysgeusia was also reported in 2 studies [21, 23]. Rhodus [21] reported dysgeusia in 34.32% of participants, while Seccia’s [23] one participant experienced this issue.

Odynophagia was reported in only 1 study [23] with this prevalence reported in this study to be 100%. Xerostomia was also reported in only 1 study [21], with this prevalence reported in this study to be 70.9%. Sensation of choking/bolus sticking in pharyngeal region was reported in 1 study [20], with this prevalence reported in this study to be 48%. Unintentional weight loss was not reported in any of the included studies.

### Critical Appraisal of Included Studies

Half of all studies were classed as moderate quality (*n* = 3; 50%), followed by strong (*n* = 2; 33.3%), and poor-quality ratings (*n* = 1; 16.66%) (see Table 5). The criteria which contributed to positive ratings primarily included the use of valid methods for the identification of fibromyalgia and dysphagia, description of the sample and setting in sufficient detail, and adopting an appropriate sample frame. Items contributing to lower ratings primarily included standard and reliable measurement for all participants, use of appropriate recruitment methods, and calculation of sample size.

**Table 5:**
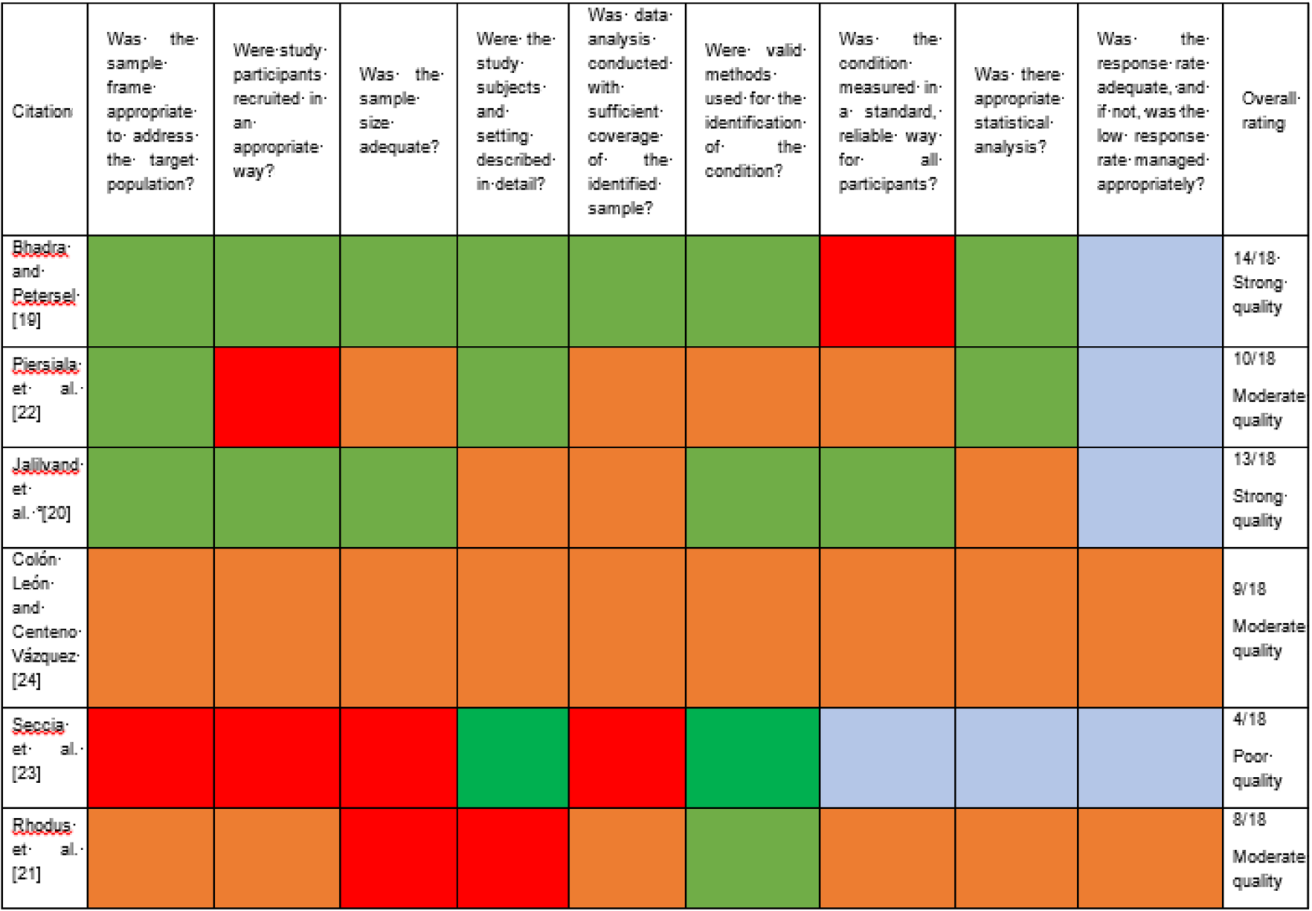
Critical appraisal of included studies

*Green* yes (2), *Red* no (0), *Orange* unclear (1), *Blue* N/A

*Poor quality* 0–6 points, *Moderate quality* 7–12 points, *Strong quality* 13–19 points

## Discussion

This systematic review represents the first attempt at synthesizing all available information regarding the prevalence of eating, drinking, and swallowing concerns among adults with fibromyalgia. Findings of this review demonstrate that limited research has been conducted on this topic, with only 6 eligible studies identified. In addition to this low number of reports, identified studies were heterogeneous in design, sample, and methodology, and of variable quality, precluding meaningful synthesis or meta-analysis in most cases. Insufficient evidence pertaining to both primary and secondary outcomes was found here to accurately answer the relevant research questions. With regards to primary outcomes, meta-analysis could only be conducted regarding the prevalence of – dysphagia (51.9%) and GERD (25.9%). While the high rates of occurrence of dysphagia and GERD in adults with fibromyalgia reported in these meta-analyses support existing research [39, 40], the impact of the results are somewhat restricted due to the limited number of studies included in each meta-analysis. Meta-analysis could not be conducted on any included secondary outcomes, as such, highlighting the scarcity of available evidence on this topic.

In addition, issues often anecdotally reported by patients to negatively impact their functioning and well-being (e.g., glossodynia or dysgeusia, odynophagia, or sensations of pharyngeal bolus sticking) were found to rarely be the primary focus of included studies here, and were given minimal attention within the published papers. Furthermore, difficulties regularly identified as core experiences of dysphagia among other clinical cohorts were often not addressed at all within studies included here (e.g., impaired, painful, or tiring mastication, aspiration/penetration, and unintentional weight loss) [41, 42]. Therefore, as these core patient issues were rarely investigated in detail, we have gathered only a small amount of new information on their nature, prevalence, or impact from the analysis here. However, evidence from other patient groups demonstrates the negative physical and psychosocial ramifications of living with such issues without appropriate clinical management, in addition to the potential for increased morbidity and mortality if they are not thoroughly addressed. Therefore, this raises the potential that due to a lack of research in this area, clinicians may not be aware of the full spectrum of swallowing difficulties that his cohort report, and therefore, patients may not receive the specialist care they require to live well with these issues, resulting in poor clinical outcomes and reduced satisfaction with care. Despite these limitations, the present systematic review suggests that eating, drinking, and swallowing issues are experienced by adults with fibromyalgia and clearly evidences the need for high quality and patient-centered research on this topic in the future in order to improve the delivery or evidence-based and empathetic care.

While the etiology of fibromyalgia-associated eating, drinking, and swallowing problems is complex and causation still not fully established, it is known that central sensitization of the nervous system plays a role in the development and maintenance of pain in fibromyalgia and has been associated with a chronic orofacial pain syndrome known as burning mouth syndrome. Burning mouth syndrome, also known as glossodynia is a chronic oropharyngeal pain syndrome characterized by a burning sensation of the oral mucosa in the absence of a specific lesion or abnormality [43]. The condition most often affects peri- and postmenopausal women and is frequently associated with dysgeusia, dysphagia, and xerostomia. The pathophysiology of burning mouth syndrome is regarded as multifactorial and complex. It is generally characterized as neuropathic in origin and has numerous contributing factors. Peripheral and central sensitization has been reported as being present in patients with burning mouth syndrome and is felt to play a critical role in the development and maintenance of the condition [44, 45]. While central sensitization may be a potential contributor to eating, drinking, and swallowing problems in patients with fibromyalgia, there are other theories within the literature that deserve mention. For example, heart rate variability has been described as a reliable biomarker for autonomic nervous system (ANS) dysfunction and has been observed as being altered in patients with a variety of pain conditions including fibromyalgia, GERD [46] and IBS [47]. The typical alterations in the ANS that have been reported include sympathetic hyperactivation and decreased parasympathetic function [48]. Adaptation to external stressors, such as a painful stimulus, has been found to be less efficient in fibromyalgia, with patients showing a higher sympathetic response with less parasympathetic recovery [49–51]. Recent studies have shown an altered intra-swallow heart rate variability with normal and effortful swallowing among healthy individuals [52, 53]. Although definitive links cannot be proven, one may hypothesize that in persons with central sensitization, associated peripheral nervous system difficulties, and an already dysregulated ANS that the autonomic changes seen during the normal swallow may be amplified and abnormal, thus leading to the experience of fibromyalgia-associated dysphagia. Further research in this area is therefore warranted to improve our understanding of this condition.

Finally, due to the complex nature of fibromyalgia, many patients are prescribed more than one medication to manage the multiple symptoms and accompanying comorbid medical and psychiatric disorders that coexist with fibromyalgia [54, 55]. Although this polypharmacy can be an effective clinical strategy for patients with fibromyalgia, these tablets may be difficult to take via the oral route if swallowing difficulties exist, and side effects can occur that can be detrimental to the patient’s health and wellbeing. Many of the medications used to treat fibromyalgia and other centralized pain syndromes have the potential to cause xerostomia, drowsiness, and gastrointestinal upset which can possibly contribute to swallowing problems, eating difficulties, and communication problems. Effective communication between the patient and their medical providers may be the most effective way to manage the potential side effects that are seen with polypharmacy. Furthermore, understanding a patient's medical history (including their prior and current medications), regular monitoring of adverse events, and discussing treatment goals can all be used to customize an effective pharmacologic regimen while attempting to minimize interactions and side effects. It is essential that medication usage and it’s contribution to/amelioration of swallowing difficulties among this group are studied further in the future to allow for development of effective management programmes for this group.

While this research represents a first step in our understanding of fibromyalgia-associated dysphagia, there are yet some tentative implications for clinical practice at this early stage. For example., it is important that clinicians become aware of the potential for dysphagia among those with fibromyalgia, and that they are prepared to listen to and value the lived experiences of their patients, advocating for onward referral to dysphagia specialists (e.g., speech and language therapists (SLTs)) were required, and contributing to and consuming future research on this topic as appropriate. In addition, as this research suggests that a cohort of people living with fibromyalgia who present to healthcare settings experience varied and currently untreated eating and drinking problems, it is essential to develop, strengthen, and advance multidisciplinary relationships among clinicians such as neurologists, rheumatologists, internists, dentists, SLTs, physiotherapists, dieticians, and other relevant professionals to improve future care delivery.

## Limitations

Although this study presents the first attempt to quantify the prevalence of eating, drinking, and swallowing issues among adults with fibromyalgia, it is prudent to note that no research is without limitations.

To begin, there has been previous variability in the diagnosis of fibromyalgia, its associated nomenclature, and diagnostic criteria. As such, the search string may have not detected certain studies which did not conform to the reference standard diagnostic criteria. Furthermore, publication bias may also add to the search detecting fewer studies than that which potentially have been conducted. However, to mitigate this potential, researchers searched several databases, conference proceedings, and reference lists to increase the coverage of the search.

Additionally, due to the limited number of studies available on this topic, sample sizes included in the analysis were small, and study designs and populations were heterogeneous. This limited the researcher’s ability to conduct large-scale meta-analysis, thus hindering researchers in adequately answering research questions.

Furthermore, FM is a diagnosis this can be observed in patients with other medical disorders that can cause eating, drinking, and swallowing problems (e.g., stroke, multiple sclerosis, rheumatologic disorders). This point is important as the present work focused only on studies done in patients with FM, however, future rigorous research is warranted to identify whether the addition of the FM diagnosis bears any additional problems when present with these co-morbid disorders. Therefore, it can co-exist in disorders that may present swallowing disorders.

Finally, many medications used to treat FM can cause side effects that may lead to eating, drinking, and swallowing problems. Most studies included in this review did not control for the use of these medications in the FM populations of interest, and as such the impact of these medications on the development and or maintenance of eating, drinking, and swallowing problems deserves further investigation.

Therefore, future studies in this area should strive to recruit larger, representative samples of people with fibromyalgia to facilitate more valid and reliable analysis of the prevalence, nature, and lived experience of fibromyalgia-associated dysphagia. It is advised that these studies investigate the potential role of co-morbid conditions or medications used for the treatment of these issues among people who are experiencing fibromyalgia-related dysphagia, using standardized diagnostic criteria, in large representative samples. This will facilitate greater understanding of common symptom profiles in this groups, with the view to develop patient-centered and evidence-based management plans into the future.

## Conclusions

This study highlighted that limited research information is available on the prevalence and nature of dysphagia among adults with fibromyalgia. However, despite the limited amount of research available, studies identified here suggest that this is an emerging field of research which requires greater attention in the future. This study also identified methodological limitations within the available literature which may impact on the generalizability of results, therefore re-emphasizing the need for future prospective and patient-led research investigating the epidemiology, nature, impact, and management of fibromylagia-related eating and swallowing problems in order to ensure that patients receive sufficient support to live well with these issues.

## References

[1] Clauw DJ (2014). Fibromyalgia: a clinical review. JAMA.

[2] Wolfe F, Ross K, Anderson J, Russell IJ, Hebert L (1995). The prevalence and characteristics of fibromyalgia in the general population. Arthritis Rheum.

[3] Wallace DJ (1997). The fibromyalgia syndrome. Ann Med.

[4] Clauw DJ, Crofford LJ (2003). Chronic widespread pain and fibromyalgia: what we know, and what we need to know. Best Pract Res Clin Rheumatol.

[5] McBeth J, Jones K (2007). Epidemiology of chronic musculoskeletal pain. Best Pract Res Clin Rheumatol.

[6] Vincent A, Lahr BD, Wolfe F, Clauw DJ, Whipple MO, Oh TH, Barton DL, St Sauver J (2013). Prevalence of fibromyalgia: a population-based study in Olmsted County, Minnesota, utilizing the Rochester epidemiology project. Arthritis Care Res.

[7] Tracey I, Bushnell MC (2009). How neuroimaging studies have challenged us to rethink: is chronic pain a disease?. J Pain.

[8] Woolf CJ (2011). Central sensitization: implications for the diagnosis and treatment of pain. Pain.

[9] Cagnie B, Coppieters I, Denecker S, Six J, Danneels L, Meeus M (2014). Central sensitization in fibromyalgia? A systematic review on structural and functional brain MRI. Semin Arthritis Rheum.

[10] Nicol AL, Sieberg CB, Clauw DJ, Hassett AL, Moser SE, Brummett CM (2016). The association between a history of lifetime traumatic events and pain severity, physical function, and affective distress in patients with chronic pain. J Pain.

[11] Williams DA, Clauw DJ (2009). Understanding fibromyalgia: lessons from the broader pain research community. J Pain.

[12] Yunus MB (2007). Fibromyalgia and overlapping disorders: the unifying concept of central sensitivity syndromes. Semin Arthritis Rheum.

[13] Panton L, Simonavice E, Williams K, Mojock C, Kim J-S, Kingsley JD, McMillan V, Mathis R (2013). Effects of class IV laser therapy on fibromyalgia impact and function in women with fibromyalgia. J Altern and Complementary Med (New York, NY).

[14] Rao SG, Bennett RM (2003). Pharmacological therapies in fibromyalgia. Best Pract Res Clin Rheumatol.

[15] Leventhal LJ (1999). Management of fibromyalgia. Ann Intern Med.

[16] Goldenberg DL (1999). Fibromyalgia syndrome a decade later: what have we learned?. Arch Intern Med.

[17] Aaron LA, Buchwald D (2001). A review of the evidence for overlap among unexplained clinical conditions. Ann internal med.

[18] Panton LB, Kingsley JD, Toole T, Cress ME, Abboud G, Sirithienthad P, Mathis R, McMillan V (2006). A comparison of physical functional performance and strength in women with fibromyalgia, age- and weight-matched controls, and older women who are healthy. Phys Ther.

[19] Bhadra P, Petersel D (2010). Medical conditions in fibromyalgia patients and their relationship to pregabalin efficacy: pooled analysis of Phase III clinical trials. Expert Opin Pharmacother.

[20] Jalilvand AD, Belle P, McNally M, Perry KA (2019). Functional gastrointestinal and neurologic disorders may alter gastroesophageal reflux disease presentation and post-operative symptomology and quality of life following nissen fundoplication. In Gastroenterology.

[21] Rhodus NL, Fricton J, Carlson P, Messner R (2003). Oral symptoms associated with fibromyalgia syndrome. J Rheumatol.

[22] Piersiala K, Akst LM, Hillel AT, Best SR (2020). Chronic pain syndromes and their laryngeal manifestations. JAMA Otolaryngol-Head & Neck Surg.

[23] Seccia TM, Rossitto G, Calò LA, Rossi GP (2015). Oral burning with dysphagia and weight loss. Medicine.

[24] Colón León RA, Centeno Vázquez MA (2021). EAT-10 and hispanics with fibromyalgia. 28th annual meeting of the dysphagia research society. Dysphagia.

[25] Liberati A, Altman DG, Tetzlaff J, Mulrow C, Gøtzsche PC, Ioannidis JPA, Clarke M, Devereaux PJ, Kleijnen J, Moher D (2009). The PRISMA statement for reporting systematic reviews and meta-analyses of studies that evaluate healthcare interventions: explanation and elaboration. BMJ (Clinical Research Ed).

[26] Gilheaney, Ó., Chadwick, A. (2021). *The Prevalence and Nature of Eating and Swallowing Problems in Adults with Fibromyalgia: A Systematic Review*. University of York. Retrieved from: https://www.crd.york.ac.uk/PROSPERO/display_record.php?RecordID=240139] 06/07/2022. 10.1007/s00455-023-10597-8 PMC10781843 37347255

[27] Zotero. (2022). Zotero - Your personal research assistant. https://www.zotero.org/

[28] Covidence. (2022). Covidence—Better systematic review management. Covidence. https://www.covidence.org/

[29] Munn Z, Tufanaru C, Aromataris E (2014). JBI’s systematic reviews: data extraction and synthesis. Am J Nurs.

[30] Joanna Briggs Institute, J. B. I. (2017). The Joanna Briggs Institute Critical Appraisal tools for use in JBI Systematic Reviews Checklist for Prevalence Studies. 7. Retrieved from: © Joanna Briggs Institute 2017 Critical Appraisal Checklist for Prevalence Studies (jbi.global) 11/05/2021.

[31] Biostat Inc., B. Inc. (2021). *Comprehensive Meta-Analysis Software (CMA)*. Retrieved from: https://www.meta-analysis.com/ 06/07/2022

[32] Melzack R (1987). The short-form McGill pain questionnaire. Pain.

[33] Wolfe F, Clauw DJ, Fitzcharles M-A, Goldenberg DL, Katz RS, Mease P, Russell AS, Russell IJ, Winfield JB, Yunus MB (2010). The American College of Rheumatology preliminary diagnostic criteria for fibromyalgia and measurement of symptom severity. Arthritis Care Res.

[34] Velanovich V, Vallance SR, Gusz JR, Tapia FV, Harkabus MA (1996). Quality of life scale for gastroesophageal reflux disease. J Am Coll Surg.

[35] Belafsky PC, Mouadeb DA, Rees CJ, Pryor JC, Postma GN, Allen J, Leonard RJ (2008). Validity and reliability of the eating assessment tool (EAT-10). Ann Otol, Rhinol Laryngol.

[36] Rhodus NL (1987). Detection and management of the dental patient with Sjörgen’s syndrome. Compendium (Newtown, Pa).

[37] Rhodus NL (1999). Sjögren’s syndrome. Quintessence International (Berlin, Germany: 1985).

[38] Melzack R, Katz J, Turk DC, Melzack R (2001). The McGill Pain Questionnaire: Appraisal and current status. Handbook of pain assessment.

[39] Wang JC, Sung FC, Men M, Wang KA, Lin CL, Kao CH (2017). Bidirectional association between fibromyalgia and gastroesophageal reflux disease: two population-based retrospective cohort analysis. Pain.

[40] De Stefano, R., Bruno, A., Muscatello, M. R. A., Cedro, C., Cicciù, A., Rullo, R., ... & Fiorillo, L. (2020). Oral health and fibromyalgia syndrome: a systemic review. Journal of Functional Morphology and Kinesiology, 5(1), 7. 10.3390/jfmk5010007 PMC7739237 33467223

[41] Hussey, J., & Gilheaney Ó., (2022). The Effect of Eating, Drinking, and Swallowing Difficulties on People with Fibromyalgia: A Qualitative Analysis of Personal Experiences. Poster presented at the *European Society for Swallowing Disorders* Conference, Leuven, Belguim, September 14–16th 2022.

[42] Gavigan, R., & Gilheaney, Ó. (2022). A Qualitative Investigation of the First-Hand Experience of Dysphagia Associated with Fibromyalgia: An Examination of Online Blogs. Poster presented at the *European Society for Swallowing Disorders* Conference, Leuven, Belguim, September 14–16th 2022.

[43] Zakrzewska JM (1995). The burning mouth syndrome remains an enigma. Pain.

[44] Feller L, Fourie J, Bouckaert M, Khammissa RAG, Ballyram R, Lemmer J (2017). Burning mouth syndrome: aetiopathogenesis and principles of management. Pain Res Manag.

[45] Basbaum AI, Bautista DM, Scherrer G, Julius D (2009). Cellular and molecular mechanisms of pain. Cell.

[46] Lee Y-C, Wang H-P, Lin L-Y, Lee B-C, Chiu H-M, Wu M-S, Chen M-F, Lin J-T (2004). Heart rate variability in patients with different manifestations of gastroesophageal reflux disease. Autonomic Neurosci.

[47] Sadowski A, Dunlap C, Lacombe A, Hanes D (2020). Alterations in heart rate variability associated with irritable bowel syndrome or inflammatory bowel disease: a systematic review and meta-analysis. Clin and Trans Gastroenterol.

[48] Cohen H, Neumann L, Kotler M, Buskila D (2001). Autonomic nervous system derangement in fibromyalgia syndrome and related disorders. Israel Med Association J.

[49] Raj SR, Brouillard D, Simpson CS, Hopman WM, Abdollah H (2000). Dysautonomia among patients with fibromyalgia: a noninvasive assessment. J Rheumatol.

[50] Reyes del Paso GA, Garrido S, Pulgar Á, Duschek S (2011). Autonomic cardiovascular control and responses to experimental pain stimulation in fibromyalgia syndrome. J Psychosom Res.

[51] Martinez-Lavin M (2007). Biology and therapy of fibromyalgia. Stress the stress response system, and fibromyalgia. Arthritis Res Ther.

[52] Yildiz M, Doma S (2021). The source of heart rhythm changes caused by swallowing. Dysphagia.

[53] Gomes LMS, Silva RG, Melo M, Silva NN, Vanderlei FM, Garner DM, de Abreu LC, Valenti VE (2016). Effects of effortful swallow on cardiac autonomic regulation. Dysphagia.

[54] Thorpe J, Shum B, Moore RA, Wiffen PJ, Gilron I (2018). Combination pharmacotherapy for the treatment of fibromyalgia in adults. Cochrane Database of Syst Rev.

[55] Boomershine CS, Crofford LJ (2009). A symptom-based approach to pharmacologic management of fibromyalgia. Nat Rev Rheumatol.

